# Association of serum phosphate levels and statin use with cardiovascular events in Japanese patients on chronic haemodialysis: a post-hoc analysis of the LANDMARK trial

**DOI:** 10.1093/ckj/sfaf151

**Published:** 2025-05-19

**Authors:** Tomohiro Saito, Masahide Mizobuchi, Akane Yamakawa, Tatsuo Kagimura, Hiroaki Ogata, Masafumi Fukagawa, Hideki Hirakata, Tadao Akizawa, Hirokazu Honda

**Affiliations:** Department of Medicine, Division of Nephrology, Showa Medical University School of Medicine, Tokyo, Japan; Division of Nephrology, Department of Internal Medicine, Showa Medical University Fujigaoka Hospital, Yokohama, Japan; The Translational Research Center for Medical Innovation, Foundation for Biomedical Research and Innovation at Kobe, Chuo-ku, Kobe, Hyogo, Japan; The Translational Research Center for Medical Innovation, Foundation for Biomedical Research and Innovation at Kobe, Chuo-ku, Kobe, Hyogo, Japan; Department of Medical Education, Showa Medical University School of Medicine, Tokyo, Japan; Department of Medicine, Showa Medical University Northern Yokohama Hospital, Yokohama, Japan; Division of Nephrology, Endocrinology and Metabolism, Tokai University School of Medicine, Isehara, Japan; Fukuoka Renal Clinic, Fukuoka, Japan; Department of Medicine, Division of Nephrology, Showa Medical University School of Medicine, Tokyo, Japan; Department of Medicine, Division of Nephrology, Showa Medical University School of Medicine, Tokyo, Japan

**Keywords:** cardiovascular events, death, haemodialysis, phosphate, statin

## Abstract

**Background:**

Statins have little beneficial effects on cardiovascular events (CVEs) in patients undergoing haemodialysis (HD) despite clinically relevant reductions in serum cholesterol levels. However, how time-dependent serum phosphate levels modify time-dependent statin use status in CVEs remains unclear. This study aimed to investigate whether statin use and time-dependent serum phosphate levels are associated with CVEs, cardiovascular death, atherosclerotic events and all-cause mortality.

**Methods:**

In this post-hoc analysis of the LANDMARK trial, we classified the Japanese patients according to statin use and serum phosphate levels and tested whether longitudinal phosphate exposure modulated the occurrence of outcomes.

**Results:**

Among 2135 patients on HD, 397 (18.6%) were prescribed statins at baseline, and 176 (8.2%) were prescribed statins during a median follow-up period of 3.2 years. Time-dependent statin administration was associated with a lower risk of all-cause death. However, there was no association between statin administration and serum phosphorus levels. Despite observing a trend towards a decreased risk of cardiovascular and atherosclerotic events for time-dependent phosphate levels <5 mg/dL during the statin prescription period, this trend was not significant. No clinical benefits of statin use on cardiovascular mortality or all-cause mortality were observed.

**Conclusion:**

Time-dependent statin use was associated with a lower risk of all-cause death. However, statin and serum phosphate levels were not significantly associated with lower risk of CVEs or mortality.

## Graphical Abstract

**Graphical Abstract:**
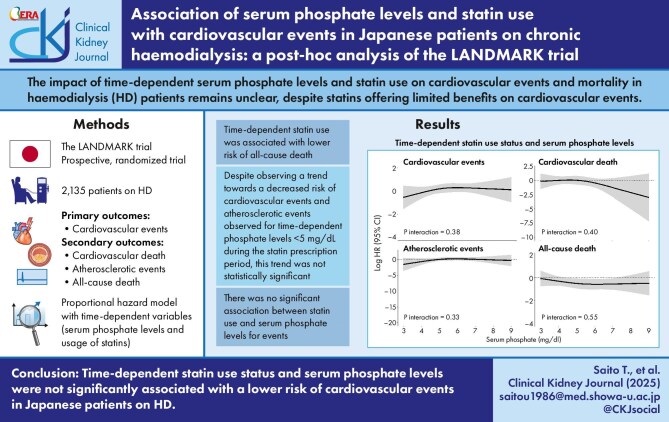

KEY LEARNING POINTS **What was known:**

- Statins have few or no beneficial effects on cardiovascular events (CVEs) in patients undergoing haemodialysis (HD).
- The association between time-dependent serum phosphate levels modify the association of time-dependent statin use status with CVEs remains unclear.

**This study adds:**

- Time-dependent statin use was associated with lower risk of all-cause death.
- Despite observing a trend towards a decreased risk of cardiovascular and atherosclerotic events for time-dependent phosphate levels <5 mg/dL during the statin prescription period, this trend was not statistically significant.
- There was no significant association between statin use and serum phosphate levels for CVEs, cardiovascular death, atherosclerotic events or all-cause death in this study population.

**Potential impact:**

- Statin use and time-dependent serum phosphate levels were not significantly associated with a lower risk of CVEs, cardiovascular death, atherosclerotic events or all-cause mortality.
- Given the exceptionally high rates of CVEs and mortality in this population compared with the general population, it may still be necessary to assess other factors for the clinical benefits of primary and secondary prevention of cardiovascular risk.

## INTRODUCTION

Cardiovascular events (CVEs) are the leading cause of morbidity and mortality in patients with end-stage kidney disease on haemodialysis (HD) [1, 2]. Statin therapy in adults with or at risk of CVEs results in consistent proportional reductions in major cardiovascular outcomes, all-cause mortality and myocardial infarction [3–6]. Despite statins having a significant low-density lipoprotein cholesterol (LDL-C)-lowering effect, randomized clinical trials (RCTs) [7–9] and high-quality meta-analyses [10–12] indicate that these drugs have little or no effect on cardiovascular outcomes in patients on HD, with several possible explanations. First, the LDL-C level was not associated with CVEs in patients undergoing HD. However, this lack of association seems unlikely, given the well-established direct causal relationship between LDL-C and atheroma [13, 14]. Second, major RCTs found limited benefit, while observational studies showed reductions in mortality and cardiac events in certain subgroups [15–17]. Furthermore, post-hoc analyses of RCTs suggest improved outcomes in specific high-risk groups [18, 19]. Therefore, we hypothesized that negative results may reflect inappropriate patient selection. Third, in addition to the established conventional risk factors for atherosclerotic CVEs, other factors, including non-traditional risk factors [e.g. chronic kidney disease–mineral and bone metabolism disorder (CKD-MBD), vascular stiffness, protein-energy wasting and calcification] and non-atherosclerotic cardiac events (e.g. arrhythmia and heart failure) play important roles in the pathophysiological characteristics of CVEs in patients on HD [1, 20, 21]. CKD-MBD occurs in patients with CKD and is associated with cardiovascular morbidity and mortality [22]; the initiating and driving force of mineral and endocrine disruptions in CKD-MBD is phosphate retention. The reason for the less effective aforementioned statins may be, at least in part, higher intracellular cholesterol production due to hyperphosphatemia [23, 24], possibly via lower membrane LDL receptor expression [25, 26].

The association between serum phosphate levels and statin therapy remains unclear. Therefore, we conducted a post-hoc analysis of the LANDMARK trial [outcome study of lanthanum carbonate (LC) compared with calcium carbonate (CC) on cardiovascular morbidity and mortality in patients with chronic kidney disease on HD] [27, 28], to clarify the association between statin therapy and serum phosphate levels on CVEs and all-cause mortality in this patient population. The primary aim of the study was to investigate whether time-dependent serum phosphate levels act as an effect modifier in the association between time-dependent statin use and CVEs and all-cause death in patients on HD.

## MATERIALS AND METHODS

### Study design and population

This was a post-hoc analysis (study approval number: 3109) of the LANDMARK trial [27–29], a multicentre, randomized, open-label, parallel comparative study of LC and CC that included patients on HD enrolled from 273 dialysis facilities across Japan who underwent randomization between March 2014 and May 2017. Participants were eligible if they were on HD for ≥3 months with phosphate binder therapy for hyperphosphatemia; had at least one risk factor for vascular calcification [≥65 years, postmenopausal or type 2 diabetes mellitus (DM)]; and had a serum intact parathyroid hormone level of ≤240 pg/mL. Patient management conformed to the Clinical Practice Guidelines for the Management of CKD–MBD published by the Japanese Society for Dialysis Therapy [30]. Target ranges of phosphate at the beginning of the first hemodialysis session of each week was 3.5–6.0 mg/dL. Details of the study design, patient eligibility and laboratory measurements have been reported previously [27–29].

The primary endpoint in both groups was the time of survival free of CVEs, including cardiovascular death, non-fatal myocardial infarction or stroke, and unstable angina. These endpoints were evaluated in a blinded fashion by an independent event evaluation committee. Follow-up visits occurred at 3, 6 and 12 months after enrolment and then each year thereafter until the end of the study, with a closeout visit.

The local institutional ethics committee approved the LANDMARK trial protocol at each site. This study was registered with ClinicalTrials.gov (NCT01578200) and umin.ac.jp (UMIN000006815), and its protocol was approved by the ethics committee of Showa Medical University Hospital (number: 3109). This study was conducted in compliance with the principles of the Declaration of Helsinki.

Because we used an anonymized dataset for research purposes, the need for written informed consent from the participants was waived. Each participant was provided with an opportunity to opt out.

### Outcomes and exposure

**Figure 1:**
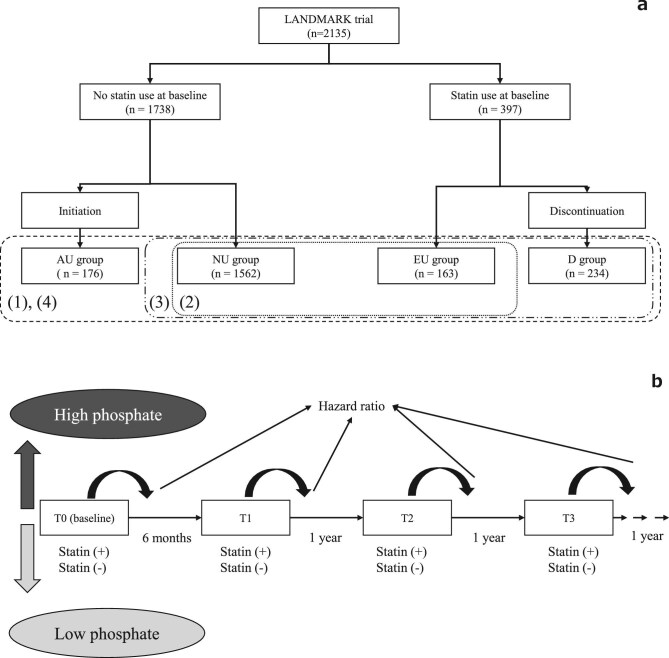
The study scheme. (**a**) (1) The participants of the LANDMARK trial were divided according to the status of statin use during the study period. The outcomes, CVEs, cardiovascular death, atherosclerotic events and all-cause death were compared between the status of statin use during the study period. (2) Those outcomes were compared between participants with statin use throughout the study period (EU group) and those without statin use throughout the study period (NU group) on outcomes stratified by a baseline serum phosphate level >5 and <5 mg/dL. (3) Using subsets of the EU, D and NU groups, we evaluated the association with baseline statin usage on time-dependent serum phosphorus concentration. Those outcomes were compared between the EU and D groups and the NU group. (4) We assessed the association with history of statin use using subsets of the EU, D, AU and NU groups. Those outcomes were compared between the EU, D and AU groups and the NU group. (**b**) Those outcomes were compared in terms of whether the interaction between phosphate and treatment affected the outcomes using Cox proportional hazards models with phosphate and statin considered as time-dependent covariates.

The primary outcome was defined as a composite of CVEs that included death due to CVEs (myocardial infarction or stroke); non-fatal myocardial infarction; non-fatal stroke; unstable angina; hospitalization for heart failure; and hospitalization for ventricular arrhythmia. Secondary outcomes were all-cause mortality, cardiovascular death and atherosclerotic events. Atherosclerotic events included cardiovascular death, ischaemic stroke, transient ischaemic attack and unstable angina. Exposure to statins was classified into four treatment strategies based on the statin prescription at baseline and during follow-up: treatment throughout the study period [ever-use (EU) group], treatment added during the study period [add-on use (AU) group], no statin treatment [non-use (NU) group] and treatment discontinued during the study period [discontinued (D) group]. The study scheme is illustrated in Fig. 1a and b.

### Statistical analysis

The demographic characteristics and baseline variables of the NU, EU, AU and D groups, which were divided based on the history of statin use during the study, were summarized as means (standard deviations) for continuous variables and numbers (percentages) for frequency variables. The cumulative incidence of CVEs, cardiovascular death, atherosclerotic events and all-cause death was calculated for each group using the Kaplan–Meier method, and the event incidence (per 100 person-years) was calculated. Log-rank tests were used to compare groups.

We estimated the nonlinear relationship using a proportional hazard model with time-dependent variables that included the presence or absence of statin use. Next, we estimated the nonlinear relationship between the hazard ratio of statin use and serum phosphorus levels in the EU and NU groups using a proportional hazard model with age, sex, smoking status, diabetes, history of cardiovascular disease, usage of renin–angiotensin system (RAS) inhibitors at baseline, and baseline values of systolic blood pressure, corrected calcium, intact parathyroid hormone, alkaline phosphatase, albumin and serum phosphorus as covariates and serum phosphorus levels measured over time as the time-dependent variable. Kidney Disease: Improving Global Outcomes (KDIGO) has issued opinion-based practice guidelines recommending aggressive treatment of hyperphosphatemia to achieve values closer to the normal range in patients undergoing HD [22]. However, a recent Japanese study showed that intensive phosphate control can slow the progression of vascular calcification [31, 32]. Another prospective Japanese cohort study further demonstrated that intensive management of hyperphosphatemia may benefit patients undergoing HD with a history of atherosclerotic cardiovascular disease or diabetic nephropathy [33]. Furthermore, associations between higher phosphate levels (>5 mg/dL) and mortality and CVEs were previously reported [23]. Thus, this relationship was evaluated by baseline serum phosphate levels stratified for >5 and <5 mg/dL.

In estimating the nonlinear relationship between hazard ratio (HR) and serum phosphate concentration, a proportional hazard model was used including an interaction term for the use of statins and serum phosphate concentration. A nonlinear relationship was estimated by fitting a restricted cubic spline of node 3 to the HR serum phosphate concentration. Additionally, we estimated the nonlinear relationship between the HR of statin use and serum phosphate concentration using a proportional hazard model with time-dependent variables that included serum phosphate concentration and the presence or absence of statin use. The assumption of proportional hazard is confirmed by the Schoenfeld individual test and Schoenfeld residual plots.

All analyses were performed using SAS version 9.4 (SAS Institute, Cary, NC, USA) and R version 4.3.1, and the significance level was set to *P* < .05 (2-tailed).

## RESULTS

### Baseline patient characteristics

This analysis included 2135 patients with baseline and follow-up data. After a median follow-up of 3.2 years, the incidence rates of CVEs were 4.8 in the LC group and 4.3 in the CC group. Baseline characteristics of the cohort (*n* = 2135) are shown in Table 1. The average age was 68.4 ± 9.6 years, 40.5% were female and the mean dialysis vintage was 6.7 ± 6.4 years. Statin therapy was discontinued in 234 (11.0%) patients in the D group. The remaining 1901 patients were divided into the NU (*n* = 1562; 73.2%), EU (*n* = 163; 7.6%) and AU groups (*n* = 176; 8.2%) (Fig. 1a). Compared with the NU group, the AU group had a shorter dialysis vintage, higher prevalence of comorbidities, and greater proportions of patients with history of myocardial infarction, coronary intervention and ischaemic stroke. Compared with the D group, the EU group had a shorter dialysis vintage, higher prevalence of comorbidities, and greater proportions of patients with history of coronary artery disease, myocardial infarction and coronary intervention. Among the four groups, 38%, 28%, 49% and 43% of patients in the AU, NU, D and EU groups were prescribed aspirin, respectively. At baseline, the mean LDL-C levels were 86.9, 76.9, 86.6 and 76.3 mg/dL in the NU, EU, AU and D groups, respectively. After 1 year, the mean LDL-C levels were 81.2 mg/dL in the AU group (mean change from baseline, –5.2 mg/dL), 76.5 mg/dL in the EU group (mean change from baseline, –2.2 mg/dL) and 77.4 mg/dL in the D group (mean change from baseline, 1.1 mg/dL) (Supplementary data, Fig. S1).

**Table 1:** Baseline characteristics of enrolled patients according to statin usage.

Item	All (*n* = 2135)	NU group (*n* = 1562)	EU group (*n* = 163)	AU group (*n* = 176)	D group (*n* = 234)
Age, years	68.4 (9.6)	68.5 (9.7)	68.2 (9.1)	67.9 (9.3)	67.5 (9.0)
Female sex, No. (%)	864 (40.5)	605 (39)	65 (40)	78 (44)	116 (50)
BMI after dialysis session, kg/m^2^	22.0 (3.7)	21.7 (3.6)	22.8 (3.4)	22.4 (3.5)	23.2 (4.3)
Current smoker, No. (%)	252 (11.8)	187 (12)	16 (9.8)	24 (14)	25 (11)
Dialysis vintage, years	6.7 (6.4)	7.1 (6.6)	5.3 (5.8)	5.6 (5.5)	5.8 (5.8)
Comorbid disease, No. (%)					
Diabetes mellitus	1198 (56.1)	806 (52)	113 (69)	118 (67)	161 (69)
Dyslipidemia	547 (25.6)	183 (12)	129 (79)	57 (32)	178 (76)
Hypertension	1735 (81.3)	1265 (81)	129 (79)	150 (85)	191 (82)
Peripheral artery disease	333 (15.6)	233 (15)	21 (13)	35 (20)	44 (19)
Past medical history					
Ischaemic heart disease					
Coronary artery disease	163 (7.6)	101 (6.5)	23 (14)	13 (7.4)	26 (11)
Myocardial infarction	97 (4.5)	46 (2.9)	18 (11)	16 (9.1)	17 (7.3)
Unstable angina	120 (5.6)	83 (5.3)	11 (6.7)	11 (6.3)	15 (6.4)
Coronary intervention	181 (8.5)	96 (6.1)	29 (18)	22 (13)	34 (15)
Cerebrovascular disease					
Ischaemic stroke	203 (9.5)	129 (8.3)	19 (12)	24 (14)	31 (13)
Haemorrhagic stroke	69 (3.2)	52 (3.3)	6 (3.7)	5 (2.8)	6 (2.6)
Transient ischaemic attack	31 (1.5)	24 (1.5)	3 (1.8)	1 (0.6)	3 (1.3)
Secondary hyperparathyroidism	867 (40.6)	625 (40)	60 (37)	83 (47)	99 (42)
Drug therapy, No. (%)					
Statin	397 (18.6)				
RAS inhibitors	918 (43.0)	650 (42)	81 (50)	81 (46)	106 (45)
Aspirin	687 (32.2)	437 (28)	70 (43)	66 (38)	114 (49)
Calcium carbonate	1583 (74.1)	1143 (73)	123 (76)	132 (75)	185 (79)
Lanthanum carbonate	628 (29.4)	468 (30)	44 (27)	45 (26)	71 (30)
Sevelamer hydrochloride	359 (16.8)	275 (18)	20 (12)	22 (13)	42 (18)
Cinacalcet	361 (16.9)	264 (17)	24 (15)	31 (18)	42 (18)
VDRAs	1435 (67.2)	1043 (67)	115 (71)	104 (59)	173 (74)
Laboratory data					
Albumin, g/dL	3.7 (0.4)	3.7 (0.4)	3.7 (0.3)	3.7 (0.4)	3.8 (0.3)
BUN before dialysis session, mg/dL	62.0 (14.0)	62.0 (14.1)	63.4 (15.1)	61.9 (14.4)	61.2 (13.0)
Cr, mg/dL	10.3 (2.3)	10.3 (2.3)	10.2 (2.1)	10.1 (2.6)	10.2 (2.3)
Corrected Ca, mg/dL	9.2 (0.7)	9.2 (0.7)	9.1 (0.6)	9.2 (0.7)	9.1 (0.6)
Phosphorus, mg/dL	5.3 (1.3)	5.3 (1.3)	5.6 (1.5)	5.4 (1.4)	5.4 (1.2)
Ca × P products, mg^2^/dL^2^	48.8 (12.4)	48.6 (12.4)	50.6 (13.4)	49.6 (12.8)	48.8 (11.0)
Intact parathyroid hormone, pg/mL	122.3 (79.6)	122.4 (81.9)	110.0 (61.4)	129.0 (77.1)	125.5 (76.5)
n-PCR, g/kg/day^a^	0.9 (0.2)	0.9 (0.2)	1.0 (0.2)	0.9 (0.2)	0.9 (0.2)
Kt/V^b^	1.6 (0.3)	1.6 (0.3)	1.6 (0.3)	1.6 (0.4)	1.5 (0.3)
Alkaline phosphatase, IU/mL	240.5 (106.2)	242.7 (110.7)	233.2 (80.3)	245.3 (104.1)	234.9 (91.7)
HbA1c, %	6.1 (1.2)	6.0 (1.1)	6.2 (1.1)	6.4 (1.0)	6.3 (1.4)
Glucose, mg/dL	131.6 (47.1)	128.4 (43.7)	139.8 (54.4)	142.0 (53.6)	137.7 (54.4)
C-reactive protein, mg/dL	0.4 (1.2)	0.4 (1.3)	0.2 (0.4)	0.4 (1.5)	0.2 (0.5)
T-C, mg/dL	156.8 (33.8)	159.1 (33.9)	149.5 (34.0)	155.1 (37.5)	148.8 (27.7)
LDL-C, mg/dL	84.8 (27.9)	86.9 (28.1)	76.9 (27.0)	86.6 (29.2)	76.3 (23.6)
HDL-C, mg/dL	48.5 (16.3)	49.0 (17.0)	48.9 (14.7)	45.5 (13.8)	47.4 (14.1)
Non-HDL-C, mg/dL	107.8 (31.4)	109.4 (31.4)	101.6 (31.8)	109.0 (35.2)	101.8 (26.6)

Continuous data presented as mean (standard deviation), categorical data presented as No. (%). *P*-values were by Wilcoxon two-sample test for continuous data and Chi-square test for categorical data.

a nPCR = C_0_/(36.3 + 5.48 **×** K_t_/V + 53.5/K_t_/V) + 0.168, where C_0_: before dialysis BUN.

b K _t_/V = −ln(C_e_/C_s_ − 0.008 **×** t_d_) + (4–3.5 **×** C_e_/C_s_) **×** ΔBW/BW, where C_s_, before dialysis BUN; C_e_, after dialysis BUN; t_d_, dialysis duration (h); ΔBW, change of body weight before and after dialysis; BW, after dialysis body weight.

BMI, body mass index; VDRAs, vitamin D receptor activators; BUN, blood urea nitrogen; Cr, creatinine; Ca, calcium; P, phosphorus; n-PCR, normalized protein catabolic rate; T-C, total cholesterol; HDL-C, high density lipoprotein cholesterol.

### Association between statin use and outcomes

**Figure 2:**
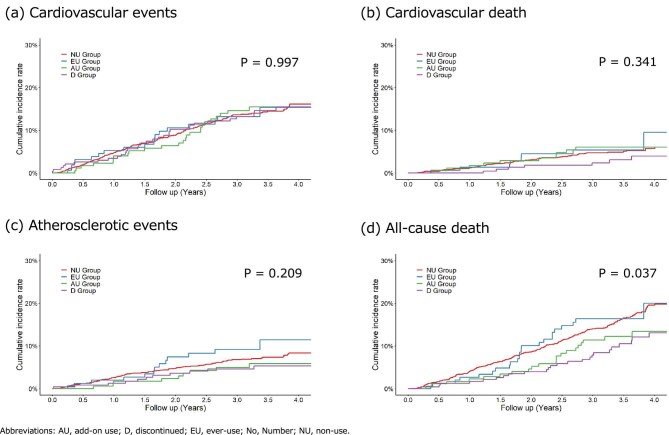
Cumulative survival rates of outcomes for each group. During the follow-up period, CVEs were observed in 281 patients, cardiovascular death in 97 patients, atherosclerotic events in 137 patients and all-cause death in 307 patients. The log-rank test revealed no significant differences between the groups for any of these four outcomes: (**a**) cardiovascular events; (**b**) cardiovascular death; (**c**) atherosclerotic events; and (**d**) all-cause death. The *P*-value of the log-rank test for the four groups are shown.

Figure 2 shows the cumulative event incidence curves for each event in the NU, EU, AU and D groups, and the results of the log-rank test for the four groups. Table 2 shows the number of events and incidence rates. Table 3 shows the HRs and 95% confidence intervals (CIs), with the NU group as the control. During the follow-up period, 281 patients developed CVEs, and the incidence rate (per 100 person-years) for each group was 4.56 for the NU group, 4.79 for the EU group, 4.52 for the AU group and 4.39 for the D group, with no difference observed between the four groups (Log-rank test *P* = .997). Cardiovascular death was observed in 97 patients, and the incidence rate (per 100 person-years) for each group was 1.51 for the NU group, 1.82 for the EU group, 1.98 for the AU group and 0.88 for the D group, with no difference observed between the four groups (Log-rank test *P* = .341). Atherosclerotic events were observed in 137 patients, and the incidence rate (per 100 person-years) for each group was 2.24 for the NU group, 3.31 for the EU group, 1.70 for the AU group and 1.54 for the D group, with no difference observed across the four groups (log-rank test *P* = .209). All-cause death was observed in 307 patients, and the incidence rate (per 100 person-years) for each group was 5.12 in the NU group, 5.23 in the EU group, 3.80 in the AU group and 3.00 in the D group, and a difference was observed in all four groups (Log-rank test *P* = .037). In the EU group, the incidence of atherosclerotic events was higher than in the NU group. In the AU group, the incidence of cardiovascular death was higher than in the NU group, and the incidence of atherosclerotic events and all-cause mortality was lower. In the D group, the incidence of CVEs, cardiovascular death, atherosclerotic events and all-cause death was lower than in the NU group, and the 95% CI for the HR for all-cause death, both adjusted and unadjusted, did not exceed 1. Table 4 shows the HRs and 95% CIs for the associations between statin use and outcomes by different statin usage. In brief, time-dependent statin use and history of statin use (EU, D and AU groups vs the NU group) were linked with a reduction in all-cause mortality. However, no significant association was identified for CVEs, cardiovascular death or atherosclerotic events.

**Table 2:** Numbers of events for statin usage and outcomes.

	NU group (*n* = 1562)		EU group (*n* = 163)		AU group (*n* = 176)		D group (*n* = 234)	
Outcome	Number	Incidence rate (per 100 PYs)	Number	Incidence rate (per 100 PYs)	Number	Incidence rate (per 100 PYs)	Number	Incidence rate (per 100 PYs)
CVEs	202	4.56	20	4.79	26	4.52	33	4.39
Cardiovascular	70	1.51	8	1.82	12	1.98	7	0.88
death								
Atherosclerotic events	101	2.24	14	3.31	10	1.70	12	1.54
All-cause	237	5.12	23	5.23	23	3.80	24	3.00
death								

PYs, person-years.

**Table 3:** HRs for study outcomes stratified by group.

			EU group (*n* = 163)	AU group (*n* = 176)	D group (*n* = 234)
Outcome		NU group (*n* = 1562)	HR (95% CI)	HR (95% CI)	HR (95% CI)
CVEs	Non-adjusted	Ref	1.04 (0.66–1.65)	1.00 (0.66–1.50)	0.97 (0.67–1.41)
	Adjusted	Ref	0.90 (0.55–1.47)	0.98 (0.64–1.51)	0.96 (0.65–1.44)
Cardiovascular death	Non-adjusted	Ref	1.21 (0.58–2.52)	1.30 (0.70–2.40)	0.57 (0.26–1.25)
	Adjusted	Ref	0.90 (0.39–2.11)	1.27 (0.66–2.44)	0.47 (0.18–1.17)
Atherosclerotic events	Non-adjusted	Ref	1.47 (0.84–2.57)	0.76 (0.40–1.45)	0.69 (0.38–1.26)
	Adjusted	Ref	1.26 (0.69–2.28)	0.77 (0.40–1.48)	0.59 (0.30–1.14)
All-cause death	Non-adjusted	Ref	1.04 (0.68–1.60)	0.73 (0.48–1.12)	0.58 (0.38–0.88)
	Adjusted	Ref	0.91 (0.57–1.45)	0.69 (0.44–1.09)	0.63 (0.40–0.99)

Adjusted HR and 95% CI were calculated using a proportional hazards model that included age, sex, smoking status, diabetes, history of cardiovascular disease, usage of RAS inhibitors at baseline, and baseline values of systolic blood pressure, corrected calcium, intact parathyroid hormone, alkaline phosphatase, albumin and serum phosphorus.

PYs, person-years; Ref, reference.

**Table 4:** HRs for the association between patterns of statin use and clinical outcomes.

	Time-dependent statin use	EU group vs NU group	EU and D group vs NU group	EU and D and AU group vs NU group
Outcome	HR (95% CI)	HR (95% CI)	HR (95% CI)	HR (95% CI)
CVEs	1.13 (0.83–1.54)	0.92 (0.56–1.51)	0.93 (0.67–1.29)	0.95 (0.72–1.26)
Cardiovascular death	0.76 (0.42–1.38)	0.89 (0.38–2.09)	0.60 (0.31–1.17)	0.85 (0.51–1.40)
Atherosclerotic events	1.07 (0.69–1.65)	1.25 (0.69–2.27)	0.83 (0.52–1.34)	0.81 (0.54–1.22)
All-cause death	0.65 (0.46–0.92)	0.92 (0.58–1.47)	0.75 (0.53–1.05)	0.73 (0.55–0.98)

The HR (95% CI) of statin use was adjusted by a proportional hazards model with age, sex, smoking status, diabetes, history of cardiovascular disease, usage of RAS inhibitors at baseline, and baseline values of systolic blood pressure, corrected calcium, intact parathyroid hormone, alkaline phosphatase, albumin and time-dependent serum phosphorus.

### Association between baseline serum phosphate level and outcomes

**Figure 3:**
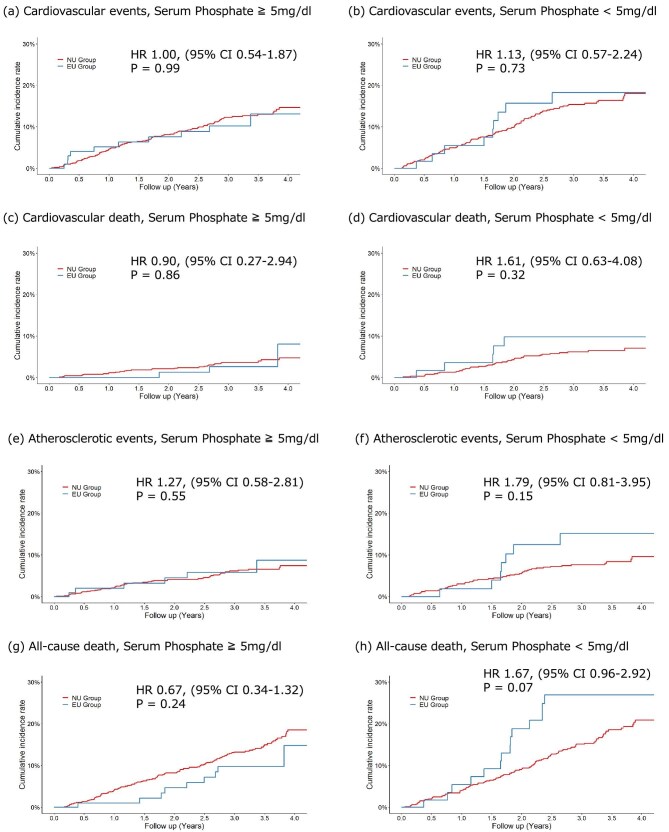
Interaction between baseline serum phosphate levels and statin use for outcomes. The findings indicate that statin use was not significantly associated with CVEs, cardiovascular death, atherosclerotic events or all-cause mortality, irrespective of baseline serum phosphate levels. (**a**, **b**) Cardiovascular events. (**c**, **d**) Cardiovascular death. (**e**, **f**) Atherosclerotic events. (**g**, **h**) All-cause death. The HR, 95% CI and *P*-value of log-rank test are shown.

We examined the association of statin usage between participants with (the EU group) and without (the NU group) statin use throughout the study period on outcomes stratified by baseline serum phosphate level >5 and <5 mg/dL. Each outcome was evaluated by dividing the baseline serum phosphate levels (Fig. 3). The HRs were as follows: CVEs, 1.00 (95% CI 0.54–1.87; *P* = .860) and 1.13 (95% CI 0.57–2.24; *P* = .860); cardiovascular death, 0.90 (95% CI 0.27–2.94; *P* = .620) and 1.61 (95% CI 0.63–4.08; *P* = .620); atherosclerotic events, 1.27 (95% CI 0.58–2.81; *P* = .180) and 1.79 (95% CI 0.81–3.95; *P* = .180); and all-cause death, 0.67 (95% CI 0.34–1.32; *P* = .860) and 1.67 (95% CI 0.96–2.92; *P* = .860).

### Association of time-dependent serum phosphate with the outcomes

Using subsets of the EU and NU groups, we estimated the spline curve of the HR for the presence or absence of statin use on serum phosphorus concentration, adjusting for age, sex, smoking status, DM and history of cardiovascular disease, with serum phosphorus concentration as a time-dependent variable (Supplementary data, Fig. S2). For all events, the HR showed a monotonic decrease up to a serum phosphate concentration of <5.0 mg/dL. However, there were no significant differences in any of the events, with *P* = .41 for CVEs, *P* = .54 for cardiovascular death, *P* = .10 for atherosclerotic events and *P* = .89 for all-cause mortality. Supplementary data, Table S1 shows the log HR and 95% CI for phosphate concentrations of 3.5, 5.0, 6.5 and 8.0 mg/dL. Similarly, using subsets of the EU, D and NU groups, we evaluated the association with baseline statin usage on serum phosphorus concentration, but no statistically significant difference was found for each event (Supplementary data, Fig. S3 and Table S2). Furthermore, we assessed the association with history of statin use using subsets of the EU, D, AU and NU groups. However, no statistically significant difference was found for each event (Supplementary data, Fig. S4 and Table S3).

### Association of time-dependent serum phosphate and statin use variations with the outcomes

**Figure 4:**
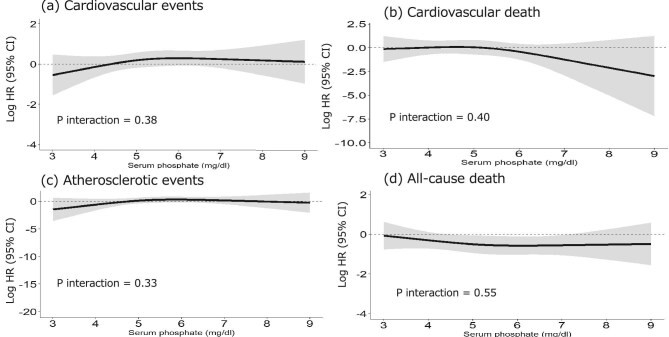
Effect of statins on outcomes for different values of time-dependent phosphate levels and statin use. Except for all-cause death, the log HR showed a monotonic decrease in serum phosphate concentration to <5.0 mg/dL in all events. There were no significant differences in any of the events. (**a**) Cardiovascular events. (**b**) Cardiovascular death. (**c**) Atherosclerotic events. (**d**) All-cause death. Models contained the interaction between time-dependent statin treatment and time-dependent serum phosphate levels and were adjusted for age, sex, smoking status, diabetes mellitus, history of cardiovascular disease, usage of RAS inhibitors at baseline, and baseline values of systolic blood pressure, corrected calcium, intact parathyroid hormone, alkaline phosphatase, albumin and serum phosphorus.

Because statin use was time-dependent, we analysed all 2135 cases, adding time-dependent statin use as a time-dependent variable to the proportional hazards model described above. The estimated spline curve of the HR for the presence or absence of statin use in relation to serum phosphorus concentration is shown in Fig. 4. Except for all-cause death, the HR showed a monotonic decrease in serum phosphate concentration to <5.0 mg/dL in all events (Table 5). There were no significant differences in any of the events, with *P* = .38 for CVEs, *P* = .40 for cardiovascular death, *P* = .33 for atherosclerotic events,and *P* = .55 for all-cause death. Table 4 shows the log HR and 95% CI for phosphate concentrations of 3.5, 5.0, 6.5 and 8.0 mg/dL.

**Table 5:** HRs for time-dependent statin usage and outcomes by serum phosphate concentration.

Outcome	Log HR for 3.5 mg/dL	Log HR for 5 mg/dL	Log HR for 6.5 mg/dL	Log HR for 8.0 mg/dL
CVEs	–0.34 (–1.10; 0.41)	0.19 (–0.19; 0.58)	0.28 (–0.10; 0.66)	0.18 (–0.56; 0.93)
Cardiovascular death	–0.05 (–1.05; 0.95)	0.05 (–0.71; 0.82)	–0.81 (–1.96; 0.34)	–2.11 (–5.03; 0.81)
Atherosclerotic events	–1.02 (–2.59; 0.54)	0.14 (–0.40; 0.68)	0.27 (–0.27; 0.82)	–0.03 (–1.26; 1.19)
All-cause death	–0.18 (–0.72; 0.35)	–0.50 (–0.95; –0.05)	–0.57 (–1.03; –0.11)	–0.52 (–1.28; 0.24)

Data presented as HR (95% CI). Models contained the interaction between time-dependent statin treatment and time-dependent serum phosphate levels and were adjusted for age, sex, smoking status, diabetes, history of cardiovascular disease, usage of RAS inhibitors at baseline, and baseline values of systolic blood pressure, corrected calcium, intact parathyroid hormone, alkaline phosphatase, albumin and serum phosphorus.

## DISCUSSION

In this study, time-dependent statin use and history of statin use (EU, D and AU groups vs the NU group) were associated with lower risk of all-cause death in Japanese patients undergoing HD. Next, we investigated the association of time-dependent serum phosphate levels and time-dependent statin therapy with CVEs and all-cause death. Despite observing a trend towards a decreased risk of CVEs and atherosclerotic events for time-dependent phosphate levels <5 mg/dL during the statin prescription period, this trend was not statistically significant and there was no significant association between statin use and serum phosphate levels for CVEs, cardiovascular death, atherosclerotic events or all-cause death in this study population. Caution must be used when interpreting this finding because of the small number of outcomes regardless of statin use (Table 2).

Statins are also effective for the primary or secondary prevention of CVEs in the general population. However, based on previous landmark RCTs [7–9], the KDIGO 2013 lipid management guideline suggested neither initiating nor discontinuing statins for the primary prevention of CVEs in patients on HD [3]. More recent lipid management guidelines provide no recommendations regarding statin therapy for patients on dialysis [5, 34]. However, in our cohort, time-dependent statin use and history of statin use (EU, D and AU groups vs the NU group) were linked to a reduction in all-cause mortality. Similar results have been reported in other retrospective studies. A recent retrospective study from Japan showed that statin therapy was associated with a reduced risk of all-cause and cardiovascular death, irrespective of the magnitude of the LDL-lowering effect [16]. South Korean nationwide claims data of incident dialysis patients showed that statin initiation was associated with a lower risk of all-cause mortality in statin-naïve patients with end-stage kidney disease [35]. Two observational studies from Taiwan showed a beneficial effect of statins in reducing the risk of all-cause mortality following myocardial infarction [15, 17]. In the A Study to Evaluate the Use of Rosuvastatin in Subjects on Regular Hemodialysis: An Assessment of Survival and Cardiovascular Events (AURORA) trial, which focused on patients undergoing HD, rosuvastatin significantly lowered LDL levels; however, this did not translate into improved outcomes [8]. However, a post-hoc analysis involving patients with DM showed reduced fatal and non-fatal cardiac events [18]. Similarly, in the Die Deutsche Diabetes Dialyse Studie (4D) trial targeting patients with diabetes on HD, atorvastatin failed to initially affect outcomes, although subsequent analysis revealed reduced fatal and non-fatal cardiac events and overall mortality in those with LDL levels >145 mg/dL, indicating a high risk [19]. Inconsistent results have been reported regarding the association between statin administration and survival in patients undergoing HD. We hypothesized that negative results in previous landmark RCTs were influenced by the inclusion of patients who were unlikely to benefit from statin therapy.

Hyperphosphatemia is associated with an increased risk of CVEs, including myocardial infarction, congestive heart failure, sudden death and peripheral arterial disease [36, 37]. Hyperphosphatemia induces endothelial dysfunction by blunting nitric oxide synthesis, which accelerates hypertension and atherosclerosis [38]. Hyperphosphatemia also drives vascular calcification via the formation and maturation of calciprotein particles and other cell-mediated calcification processes by sodium-phosphate co-transporters [39, 40]. A recent RCT targeting patients with hyperphosphatemia on HD showed that strict phosphate control using phosphate binders reduced the progression of coronary arterial calcification compared with non-strict phosphate control [32]. Recently, hyperphosphatemia has been reported to be associated with lipid metabolism. Another study revealed the mechanism underlying the link between phosphate and cholesterol metabolism [24]. Phosphate incorporated into vascular smooth muscle cells modulates α-mannosidase II-mediated glycosylation status of sterol regulatory element binding protein 2 (SREBP2) cleavage-activating protein, a sensor of intracellular cholesterol pool, which activates a transcription factor, SREBP2, and enhances transcription of the target genes of SREBP2, including HMG-CoA reductase and LDL-C receptor. These findings may explain the clinical observations and suggest a possible link between phosphate and atherosclerosis. Since Niemann-Pick C1-like 1, an intestinal cholesterol transporter, is one of the targets of SREBP2, hyperphosphatemia may result in increased intestinal cholesterol absorption [41, 42]. A recent clinical study revealed an independent association between serum phosphate and campesterol levels, a marker of intestinal cholesterol absorption, in patients with kidney failure undergoing HD [43]. These reports have suggested that the protective effect of statins on cardiovascular outcomes may be attenuated in subgroups of patients with increased cholesterol absorption and elevated serum phosphate levels. However, while the potential mechanisms linking phosphate and cholesterol metabolism are intriguing, they remain largely speculative.

Our study showed that time-dependent statin use and time-dependent phosphate levels were not associated with reduced outcomes. Massy *et al*. showed that the association between statin therapy and CVEs and all-cause mortality appeared to differ depending on time-dependent serum phosphate concentrations in a post-hoc analysis of the AURORA and 4D trials [23]. The lower the serum phosphate level, the greater the protective association between statin therapy for CVEs and all-cause mortality. However, the statistical interaction was only significant for the treatment effects on all-cause mortality. Compared with the major trials (e.g. 4D, AURORA and Study of Heart and Renal Protection [SHARP]), our cohort had lower event rate older patients, lower serum albumin levels, fewer patients with diabetic nephropathy and lower incidence of CVEs—all factors that may help explain the null results related to the time-dependent phosphate levels. The lack of statistically significant findings may be due to the low event rates and limited statistical power, as well as the small effect size in this population. Therefore, it may not have had sufficient power to detect differences in outcomes [27, 28]. When the analyses were repeated using the baseline serum phosphate concentrations analysed (phosphate level <5 or ≥5 mg/dL), the findings were directionally similar, although the test for statistical interaction was nonspecific for either outcome. The results of the latter analyses did not differ after adjusting for time-dependent LDL concentrations. Notably, the findings could not be replicated in the 4D trial. Therefore, the lack of clear significant results prevents a definitive conclusion. Given the null findings of this study, future research should focus on exploring alternative phosphate management strategies and their impact on cardiovascular outcomes in haemodialysis patients. In a post-hoc analysis of RAS inhibitors using the same cohort [44], there was an association between serum phosphate levels and CVEs. However, it should be noted that the HR for CVEs did not increase even when serum phosphate levels increased in this study. Randomized controlled trials evaluating the efficacy of stricter phosphate control [32, 45] or the use of novel phosphate binders [46] with statin use could provide insights into optimizing treatment protocols. Furthermore, a recent Japanese nationwide prospective cohort study reported that intensive phosphate management may provide greater benefits in patients with a history of atherosclerotic cardiovascular disease or diabetic nephropathy. Additionally, because the extent of benefit from reduced phosphate levels depends on serum albumin in the oldest-old population, a multifaceted approach that considers age and nutritional status may be needed to manage serum phosphate optimally [33]. In our cohort, 16.8% of patients were using sevelamer hydrochloride. This phosphate binder lowers cholesterol by binding bile acids in the gut, which reduces their reabsorption. This process leads to a decrease in serum LDL-C and total cholesterol levels [47, 48]. However, we did not perform a sub-analysis due to the small number of patients. Hence, future studies should adopt a multi-layered design that (i) stratifies patients by age, diabetic status and nutritional status; (ii) integrates advanced calcification imaging (e.g. coronary CT Agatston score) together with time-weighted mean phosphate levels; and (iii) conducts randomized factorial trials that compare (a) intensive phosphate control (target <5 mg/dL) with vs without statin therapy and (b) lipophilic vs hydrophilic statins. Such an approach would better stratify cardiovascular risk in this population and may yield more targeted therapeutic approaches.

This study has several limitations. First, as this was a sub-analysis of the LANDMARK trial [27, 28], patients were not randomly assigned to the statin use or non-user groups. The time of initiation, duration, type, dose of preprocedural statin therapy, adverse effects, statin adherence and the reasons for statin withdrawal after dialysis initiation were not well documented. Unlike hydrophilic statins, lipophilic statins may inhibit the enzymatic activity of UbiA prenyltransferase domain-containing protein 1, an enzyme which plays an important role in vitamin K2 synthesis [49]. Therefore, lipophilic statins may accelerate arterial calcification by depleting vascular vitamin K2 levels, increasing uncarboxylated matrix-Gla protein levels [50]. Additionally, the variability in adherence to prescribed treatments and its potential impact on serum phosphate levels, lipid profiles and cardiovascular outcomes could not be evaluated. This variability introduces a potential confounding factor which we could not account for in this study. Second, dietary protein intake and phosphate binder dosage were not incorporated into the covariates. Dietary phosphate intake significantly influences serum phosphate levels and, along with binder dosage, can impact cardiovascular outcomes. However, these data were not collected in the LANDMARK trial, underscoring the need for future studies to include such variables to better understand their role in statin use and cardiovascular risk. Third, our results are based on a single ethnic population from a single country. To enhance the applicability of our findings, future research should aim to validate these results in diverse populations, including those from multi-ethnic cohorts and varied geographic regions. Conducting international multicentre studies or meta-analyses pooling data from different settings would be valuable in confirming the robustness of our observations across heterogeneous patient populations.

In conclusion, this study showed that time-dependent statin use was associated with a lower risk of all-cause death in Japanese patients on HD. However, statin use and time-dependent serum phosphate levels were not significantly associated with a lower risk of CVEs, cardiovascular death, atherosclerotic events or all-cause mortality.

## Supplementary Material

sfaf151_Supplemental_Files

## Data Availability

The data underlying this article will be shared on reasonable request to the corresponding author.

## References

[1] Wanner C, Amann K, Shoji T. The heart and vascular system in dialysis. Lancet 2016;388:276–84. 10.1016/S0140-6736(16)30508-6 27226133

[2] Cozzolino M, Mangano M, Stucchi A et al. Cardiovascular disease in dialysis patients. Nephrol Dial Transplant 2018;33:iii28–34. 10.1093/ndt/gfy174 30281132 PMC6168816

[3] Wanner C, Tonelli M. KDIGO Clinical Practice Guideline for Lipid Management in CKD: summary of recommendation statements and clinical approach to the patient. Kidney Int 2014;85:1303–9. 10.1038/ki.2014.31 24552851

[4] Chou R, Dana T, Blazina I et al. Statins for prevention of cardiovascular disease in adults: evidence report and systematic review for the US Preventive Services Task Force. JAMA 2016;316:2008–24. 10.1001/jama.2015.15629 27838722

[5] Grundy SM, Stone NJ, Bailey AL et al. 2018 AHA/ACC/AACVPR/AAPA/ABC/ACPM/ADA/AGS/APhA/ASPC/NLA/PCNA Guideline on the management of blood cholesterol: a report of the American College of Cardiology/American Heart Association Task Force on Clinical Practice Guidelines. Circulation 2019;139:e1082–143. 30586774 10.1161/CIR.0000000000000625 PMC7403606

[6] Mach F, Baigent C, Catapano AL et al. 2019 ESC/EAS guidelines for the management of dyslipidaemias: lipid modification to reduce cardiovascular risk. Eur Heart J 2020;41:111–88. 10.1093/eurheartj/ehz455 31504418

[7] Wanner C, Krane V, März W et al. Atorvastatin in patients with type 2 diabetes mellitus undergoing hemodialysis. N Engl J Med 2005;353:238–48. 10.1056/NEJMoa043545 16034009

[8] Fellström BC, Jardine AG, Schmieder RE et al. Rosuvastatin and cardiovascular events in patients undergoing hemodialysis. N Engl J Med 2009;360:1395–407. 10.1056/NEJMoa0810177 19332456

[9] Baigent C, Landray MJ, Reith C et al. The effects of lowering LDL cholesterol with simvastatin plus ezetimibe in patients with chronic kidney disease (Study of Heart and Renal Protection): a randomised placebo-controlled trial. Lancet 2011;377:2181–92. 10.1016/S0140-6736(11)60739-3 21663949 PMC3145073

[10] Ghayda RA, Lee JY, Yang JW et al. The effect of statins on all-cause and cardiovascular mortality in patients with non-dialysis chronic kidney disease, patients on dialysis, and kidney transplanted recipients: an umbrella review of meta-analyses. Eur Rev Med Pharmacol Sci 2021;25:2696–710. 33829456 10.26355/eurrev_202103_25433

[11] Messow CM, Isles C. Meta-analysis of statins in chronic kidney disease: who benefits? QJM 2017;110:493–500. 10.1093/qjmed/hcx040 28340216

[12] Palmer SC, Navaneethan SD, Craig JC et al. HMG CoA reductase inhibitors (statins) for dialysis patients. Cochrane Database Syst Rev 2013;2013:Cd004289. 24022428 10.1002/14651858.CD004289.pub5 PMC10754478

[13] Silverman MG, Ference BA, Im K et al. Association between lowering LDL-C and cardiovascular risk reduction among different therapeutic interventions: a systematic review and meta-analysis. JAMA 2016;316:1289–97. 10.1001/jama.2016.13985 27673306

[14] Collins R, Reith C, Emberson J et al. Interpretation of the evidence for the efficacy and safety of statin therapy. Lancet 2016;388:2532–61. 10.1016/S0140-6736(16)31357-5 27616593

[15] Chung CM, Lin MS, Chang CH et al. Moderate to high intensity statin in dialysis patients after acute myocardial infarction: a national cohort study in Asia. Atherosclerosis 2017;267:158–66. 10.1016/j.atherosclerosis.2017.09.018 28985950

[16] Funamizu T, Iwata H, Chikata Y et al. A prognostic merit of statins in patients with chronic hemodialysis after percutaneous coronary intervention-a 10-year follow-up Study. J Clin Med 2022;11:390. 10.3390/jcm11020390 35054080 PMC8780570

[17] Li YR, Tsai SS, Lin YS et al. Moderate- to high-intensity statins for secondary prevention in patients with type 2 diabetes mellitus on dialysis after acute myocardial infarction. Diabetol Metab Syndr 2017;9:71. 10.1186/s13098-017-0272-7 28932290 PMC5605978

[18] Holdaas H, Holme I, Schmieder RE et al. Rosuvastatin in diabetic hemodialysis patients. J Am Soc Nephrol 2011;22:1335–41. 10.1681/ASN.2010090987 21566054 PMC3137581

[19] März W, Genser B, Drechsler C et al. Atorvastatin and low-density lipoprotein cholesterol in type 2 diabetes mellitus patients on hemodialysis. Clin J Am Soc Nephrol 2011;6:1316–25. 10.2215/CJN.09121010 21493741 PMC3109927

[20] Kassimatis TI, Goldsmith DJ. Statins in chronic kidney disease and kidney transplantation. Pharmacol Res 2014;88:62–73. 10.1016/j.phrs.2014.06.011 24995940

[21] Lodebo BT, Shah A, Kopple JD. Is it important to prevent and treat protein-energy wasting in chronic kidney disease and chronic dialysis patients? J Ren Nutr 2018;28:369–79. 10.1053/j.jrn.2018.04.002 30057212

[22] KDIGO 2017 clinical practice guideline update for the diagnosis, evaluation, prevention, and treatment of chronic kidney disease-mineral and bone disorder (CKD-MBD). Kidney Int Suppl 2017;7:1–59. 10.1016/j.kisu.2017.04.001 PMC6340919 30675420

[23] Massy ZA, Merkling T, Wagner S et al. Association of serum phosphate with efficacy of statin therapy in hemodialysis patients. Clin J Am Soc Nephrol 2022;17:546–54. 10.2215/CJN.12620921 35236715 PMC8993469

[24] Zhou C, He Q, Gan H et al. Hyperphosphatemia in chronic kidney disease exacerbates atherosclerosis via a mannosidases-mediated complex-type conversion of SCAP N-glycans. Kidney Int 2021;99:1342–53. 10.1016/j.kint.2021.01.016 33631226

[25] Dhingra S, Bansal MP. Hypercholesterolemia and LDL receptor mRNA expression: modulation by selenium supplementation. Biometals 2006;19:493–501. 10.1007/s10534-005-5393-z 16937255

[26] Grundmann SM, Schutkowski A, Berger C et al. High-phosphorus diets reduce aortic lesions and cardiomyocyte size and modify lipid metabolism in Ldl receptor knockout mice. Sci Rep 2020;10:20748. 10.1038/s41598-020-77509-w 33247205 PMC7695849

[27] Ogata H, Fukagawa M, Hirakata H et al. Design and baseline characteristics of the LANDMARK study. Clin Exp Nephrol 2017;21:531–7. 10.1007/s10157-016-1310-8 27405619 PMC5556131

[28] Ogata H, Fukagawa M, Hirakata H et al. Effect of treating hyperphosphatemia with lanthanum carbonate vs calcium carbonate on cardiovascular events in patients with chronic kidney disease undergoing hemodialysis: the LANDMARK randomized clinical trial. JAMA 2021;325:1946–54. 10.1001/jama.2021.4807 34003226 PMC8132143

[29] Ogata H, Fukagawa M, Hirakata H et al. Effect of lanthanum carbonate and calcium carbonate on the progression of coronary artery calcification among hemodialysis patients with vascular calcification risk: a randomized controlled trial. Clin Exp Nephrol 2022;26:1223–32. 10.1007/s10157-022-02270-5 36064876

[30] Fukagawa M, Yokoyama K, Koiwa F et al. Clinical practice guideline for the management of chronic kidney disease-mineral and bone disorder. Ther Apher Dial 2013;17:247–88. 10.1111/1744-9987.12058 23735142

[31] Fujii H, Kono K, Nakai K et al. Effects of lanthanum carbonate on coronary artery calcification and cardiac abnormalities after initiating hemodialysis. Calcif Tissue Int 2018;102:310–20. 10.1007/s00223-017-0347-3 29058057

[32] Isaka Y, Hamano T, Fujii H et al. Optimal phosphate control related to coronary artery calcification in dialysis patients. J Am Soc Nephrol 2021;32:723–35. 10.1681/ASN.2020050598 33547218 PMC7920180

[33] Goto S, Hamano T, Fujii H et al. The benefit of reduced serum phosphate levels depends on patient characteristics: a nationwide prospective cohort study. Clin Kidney J 2024;17:sfae263. 10.1093/ckj/sfae263 39385948 PMC11462437

[34] Arnett DK, Blumenthal RS, Albert MA et al. 2019 ACC/AHA guideline on the primary prevention of cardiovascular disease: a report of the American College of Cardiology/American Heart Association Task Force on Clinical Practice Guidelines. Circulation 2019;140:e596–646. 30879355 10.1161/CIR.0000000000000678 PMC7734661

[35] Kim JE, Park S, Kim MS et al. Statin initiation and all-cause mortality in incident statin-naïve dialysis patients. Atherosclerosis 2021;337:59–65. 10.1016/j.atherosclerosis.2021.08.026 34429195

[36] Shimamoto S, Yamada S, Hiyamuta H et al. Association of serum phosphate concentration with the incidence of intervention for peripheral artery disease in patients undergoing hemodialysis: 10-year outcomes of the Q-Cohort Study. Atherosclerosis 2020;304:22–9. 10.1016/j.atherosclerosis.2020.04.022 32563735

[37] Hiyamuta H, Yamada S, Taniguchi M et al. Association of hyperphosphatemia with an increased risk of sudden death in patients on hemodialysis: ten-year outcomes of the Q-Cohort Study. Atherosclerosis 2021;316:25–31. 10.1016/j.atherosclerosis.2020.11.020 33260008

[38] Shuto E, Taketani Y, Tanaka R et al. Dietary phosphorus acutely impairs endothelial function. J Am Soc Nephrol 2009;20:1504–12. 10.1681/ASN.2008101106 19406976 PMC2709683

[39] Yamada S, Leaf EM, Chia JJ et al. PiT-2, a type III sodium-dependent phosphate transporter, protects against vascular calcification in mice with chronic kidney disease fed a high-phosphate diet. Kidney Int 2018;94:716–27. 10.1016/j.kint.2018.05.015 30041812 PMC6211801

[40] Kuro OM. Klotho and calciprotein particles as therapeutic targets against accelerated ageing. Clin Sci (Lond) 2021;135:1915–27. 10.1042/CS20201453 34374422 PMC8355631

[41] Gévry N, Schoonjans K, Guay F et al. Cholesterol supply and SREBPs modulate transcription of the Niemann-Pick C-1 gene in steroidogenic tissues. J Lipid Res 2008;49:1024–33. 10.1194/jlr.M700554-JLR200 18272928

[42] Pramfalk C, Jiang ZY, Cai Q et al. HNF1alpha and SREBP2 are important regulators of NPC1L1 in human liver. J Lipid Res 2010;51:1354–62. 10.1194/jlr.M900274-JLR200 20460578 PMC3035498

[43] Okute Y, Shoji T, Shimomura N et al. Serum phosphate as an independent factor associated with cholesterol metabolism in patients undergoing hemodialysis: a cross-sectional analysis of the DREAM cohort. Nephrol Dial Transplant 2023;38:1002–8. 10.1093/ndt/gfac222 35869969

[44] Saito Y, Ito H, Fukagawa M et al. Effect of renin-angiotensin system inhibitors on cardiovascular events in hemodialysis patients with hyperphosphatemia: a post hoc analysis of the LANDMARK trial. Ther Apher Dial 2024;28:192–205. 10.1111/1744-9987.14080 37921027

[45] Shimizu M, Fujii H, Kono K et al. Clinical implication of consistently strict phosphate control for coronary and valvular calcification in incident patients undergoing hemodialysis. J Atheroscler Thromb 2023;30:1568–79. 10.5551/jat.64159 36990726 PMC10627770

[46] Block GA, Rosenbaum DP, Yan A et al. Efficacy and safety of tenapanor in patients with hyperphosphatemia receiving maintenance hemodialysis: a randomized phase 3 trial. J Am Soc Nephrol 2019;30:641–52. 10.1681/ASN.2018080832 30846557 PMC6442342

[47] Basutkar RS, Varghese R, Mathew NK et al. Systematic review and meta-analysis of potential pleiotropic effects of sevelamer in chronic kidney disease: beyond phosphate control. Nephrology 2022;27:337–54. 10.1111/nep.14011 34882904

[48] Brandenburg VM, Schlieper G, Heussen N et al. Serological cardiovascular and mortality risk predictors in dialysis patients receiving sevelamer: a prospective study. Nephrol Dial Transplant 2010;25:2672–9. 10.1093/ndt/gfq053 20172849

[49] Hirota Y, Nakagawa K, Sawada N et al. Functional characterization of the vitamin K2 biosynthetic enzyme UBIAD1. PLoS One 2015;10:e0125737. 10.1371/journal.pone.0125737 25874989 PMC4398444

[50] Schurgers LJ, Uitto J, Reutelingsperger CP. Vitamin K-dependent carboxylation of matrix Gla-protein: a crucial switch to control ectopic mineralization. Trends Mol Med 2013;19:217–26. 10.1016/j.molmed.2012.12.008 23375872

