# Supplementary material for: Association of serum phosphate levels and statin use with cardiovascular events in Japanese patients on chronic haemodialysis: a post-hoc analysis of the LANDMARK trial
Source: Clin Kidney J. 2025 May 19;18(6):sfaf151. doi: 10.1093/ckj/sfaf151 (PMC12164752; doi:10.1093/ckj/sfaf151)
Supplement: sfaf151_Supplemental_Files [file sfaf151_supplemental_files.zip › Supplement_figure_legends_2024_1215.docx]

**Supplemental Figure 1.** Mean cholesterol during follow-up. (a) Total cholesterol, (b) low-density lipid-cholesterol, (c) high-density lipid-cholesterol, and (d) non-high-density lipid-cholesterol according to statin usage. After one-year, total cholesterol and low-density lipid-cholesterol levels decreased in the AU and EU groups, while they slightly increased in the D group. Abbreviations: AU, add-on use; D, discontinued; EU, ever-use; HDL-C, high-density lipid-cholesterol; LDL-C, low-density lipid-cholesterol; non-HDL-C, non-high-density lipid-cholesterol; NU, non-user; T-C, Total cholesterol.

**Supplemental Figure 2.** Effect of statins on outcomes for different values of time-dependent phosphate levels. (a) Cardiovascular events. (b) Cardiovascular death. (c) Atherosclerotic events. (d) All-cause death. Although no significant differences were observed in any of the events, the log hazard ratio showed a monotonic decrease for serum phosphate concentrations below 5.0 mg/dL. Models contained the interaction between statin treatment and serum phosphate levels and were adjusted for age, sex, smoking status, diabetes, history of cardiovascular disease, usage of renin-angiotensin system inhibitors at baseline, and baseline values of systolic blood pressure, corrected calcium, intact parathyroid hormone, alkaline phosphatase, albumin, and serum phosphorus. Abbreviations: HR, hazard ratio; CI, confidence interval.

**Supplemental Figure 3.** Effect of baseline statin usage on outcomes for different values of time-dependent phosphate levels. (a) Cardiovascular events. (b) Cardiovascular death. (c) Atherosclerotic events. (d) All-cause death. Although no significant differences were observed in any of the events, the log hazard ratio showed a monotonic decrease for serum phosphate concentrations below 5.0 mg/dL. Models contained the interaction between statin treatment and serum phosphate levels and were adjusted for age, sex, smoking status, diabetes, history of cardiovascular disease, usage of renin-angiotensin system inhibitors at baseline, and baseline values of systolic blood pressure, corrected calcium, intact parathyroid hormone, alkaline phosphatase, albumin, and serum phosphorus. Abbreviations: HR, hazard ratio; CI, confidence interval.

**Supplemental Figure 4.** Effect of history of statin usage on outcomes for different values of time-dependent phosphate levels. (a) Cardiovascular events. (b) Cardiovascular death. (c) Atherosclerotic events. (d) All-cause death. There were no significant differences were　observed in any of the events. Models contained the interaction between statin treatment and serum phosphate levels and were adjusted for age, sex, smoking status, diabetes, history of cardiovascular disease, usage of renin-angiotensin system inhibitors at baseline, and baseline values of systolic blood pressure, corrected calcium, intact parathyroid hormone, alkaline phosphatase, albumin, and serum phosphorus. Abbreviations: HR, hazard ratio; CI, confidence interval.
