# Supplementary material for: Association of serum phosphate levels and statin use with cardiovascular events in Japanese patients on chronic haemodialysis: a post-hoc analysis of the LANDMARK trial
Source: Clin Kidney J. 2025 May 19;18(6):sfaf151. doi: 10.1093/ckj/sfaf151 (PMC12164752; doi:10.1093/ckj/sfaf151)
Supplement: sfaf151_Supplemental_Files [file sfaf151_supplemental_files.zip › Sup_Table_1_2024_1215.docx]

Supplementary Table 1. Hazard ratios for statin usage and outcomes by serum phosphate concentration.

| Outcome | log HR for 3.5 mg/dL | log HR for 5 mg/dL | log HR for 6.5 mg/dL | log HR for 8.0 mg/dL |
| --- | --- | --- | --- | --- |
| Cardiovascular events | -1.07 (-2.71; 0.58) | 0.04 (-0.59; 0.66) | 0.14 (-0.46; 0.74) | -0.17 (-1.42; 1.08) |
| Cardiovascular death | -0.16 (-1.89; 1.57) | 0.41 (-0.68; 1.49) | -0.85 (-2.87; 1.17) | -2.85 (-8.14; 2.45) |
| Atherosclerotic events | -6.42 (-13.44; 0.61) | 0.30 ( -0.72; 1.31) | 0.15 ( -0.96; 1.26) | -2.78 ( -6.67; 1.11) |
| All-cause death | -0.11 (-0.91; 0.68) | 0.01 (-0.60; 0.61) | -0.10 (-0.71; 0.51) | -0.30 (-1.39; 0.80) |

Data presented hazard ratio (HR) (95% confidence interval). Models contained the interaction between statin treatment and time-dependent serum phosphate levels and were adjusted for age, sex, smoking status, diabetes, history of cardiovascular disease, usage of renin-angiotensin system inhibitors at baseline, and baseline values of systolic blood pressure, corrected calcium, intact parathyroid hormone, alkaline phosphatase, albumin, and serum phosphorus.
