# Supplementary material for: Association of serum phosphate levels and statin use with cardiovascular events in Japanese patients on chronic haemodialysis: a post-hoc analysis of the LANDMARK trial
Source: Clin Kidney J. 2025 May 19;18(6):sfaf151. doi: 10.1093/ckj/sfaf151 (PMC12164752; doi:10.1093/ckj/sfaf151)
Supplement: sfaf151_Supplemental_Files [file sfaf151_supplemental_files.zip › Sup_Table_2_2024_1215.docx]

Supplementary Table 2. Hazard ratios for baseline statin usage and outcomes by serum phosphate concentration, comparing the combined EU and D groups to the NU group.

| Outcome | log HR for 3.5 mg/dL | log HR for 5 mg/dL | log HR for 6.5 mg/dL | log HR for 8.0 mg/dL |
| --- | --- | --- | --- | --- |
| Cardiovascular events | -0.44 (-1.23; 0.34) | -0.02 (-0.44; 0.39) | 0.05 (-0.37; 0.46) | -0.03 (-0.83; 0.77) |
| Cardiovascular death | -0.63 (-1.89; 0.64) | -0.34 (-1.18; 0.50) | -0.53 (-1.42; 0.36) | -0.92 (-2.78; 0.94) |
| Atherosclerotic events | -1.95 (-4.04; 0.14) | 0.21 (-0.38; 0.79) | -0.31 (-1.10; 0.48) | -1.94 (-4.19; 0.31) |
| All-cause death | -0.62 (-1.28; 0.05) | -0.17 (-0.60; 0.26) | -0.14 (-0.58; 0.29) | -0.29 (-1.06; 0.48) |

Data presented hazard ratio (HR) (95% confidence interval). Models contained the interaction between baseline statin treatment and time-dependent serum phosphate levels and were adjusted for age, sex, smoking status, diabetes, history of cardiovascular disease, usage of renin-angiotensin system inhibitors at baseline, and baseline values of systolic blood pressure, corrected calcium, intact parathyroid hormone, alkaline phosphatase, albumin, and serum phosphorus.
