# Supplementary material for: Association of serum phosphate levels and statin use with cardiovascular events in Japanese patients on chronic haemodialysis: a post-hoc analysis of the LANDMARK trial
Source: Clin Kidney J. 2025 May 19;18(6):sfaf151. doi: 10.1093/ckj/sfaf151 (PMC12164752; doi:10.1093/ckj/sfaf151)
Supplement: sfaf151_Supplemental_Files [file sfaf151_supplemental_files.zip › Sup_Table_3_2024_1215.docx]

Supplementary Table 3. Hazard ratios for outcomes based on history of statin usage and serum phosphate concentration, comparing the combined EU, AU, and D groups to the NU group.

| Outcome | log HR for 3.5 mg/dL | log HR for 5 mg/dL | log HR for 6.5 mg/dL | log HR for 8.0 mg/dL |
| --- | --- | --- | --- | --- |
| Cardiovascular events | -0.07 (-0.61; 0.48) | -0.07 (-0.43; 0.29) | -0.03 (-0.39; 0.33) | 0.03 (-0.66; 0.71) |
| Cardiovascular death | 0.07 (-0.71; 0.85) | -0.17 (-0.82; 0.48) | -0.36 (-1.05; 0.33) | -0.53 (-1.89; 0.82) |
| Atherosclerotic events | -0.69 (-1.82; 0.44) | -0.11 (-0.62; 0.39) | -0.14 (-0.67; 0.40) | -0.42 (-1.59; 0.76) |
| All-cause death | -0.33 (-0.79; 0.12) | -0.31 (-0.68; 0.06) | -0.30 (-0.68; 0.08) | -0.29 (-0.93; 0.36) |

Data presented hazard ratio (HR) (95% confidence interval). Models contained the interaction between baseline statin treatment and time-dependent serum phosphate levels and were adjusted for age, sex, smoking status, diabetes, history of cardiovascular disease, usage of renin-angiotensin system inhibitors at baseline, and baseline values of systolic blood pressure, corrected calcium, intact parathyroid hormone, alkaline phosphatase, albumin, and serum phosphorus.
